# Supplementary figures and images for: Automatic generation of objective footprint outlines
Source: PeerJ. 2019 Jun 27;7:e7203. doi: 10.7717/peerj.7203 (PMC6599673; doi:10.7717/peerj.7203)

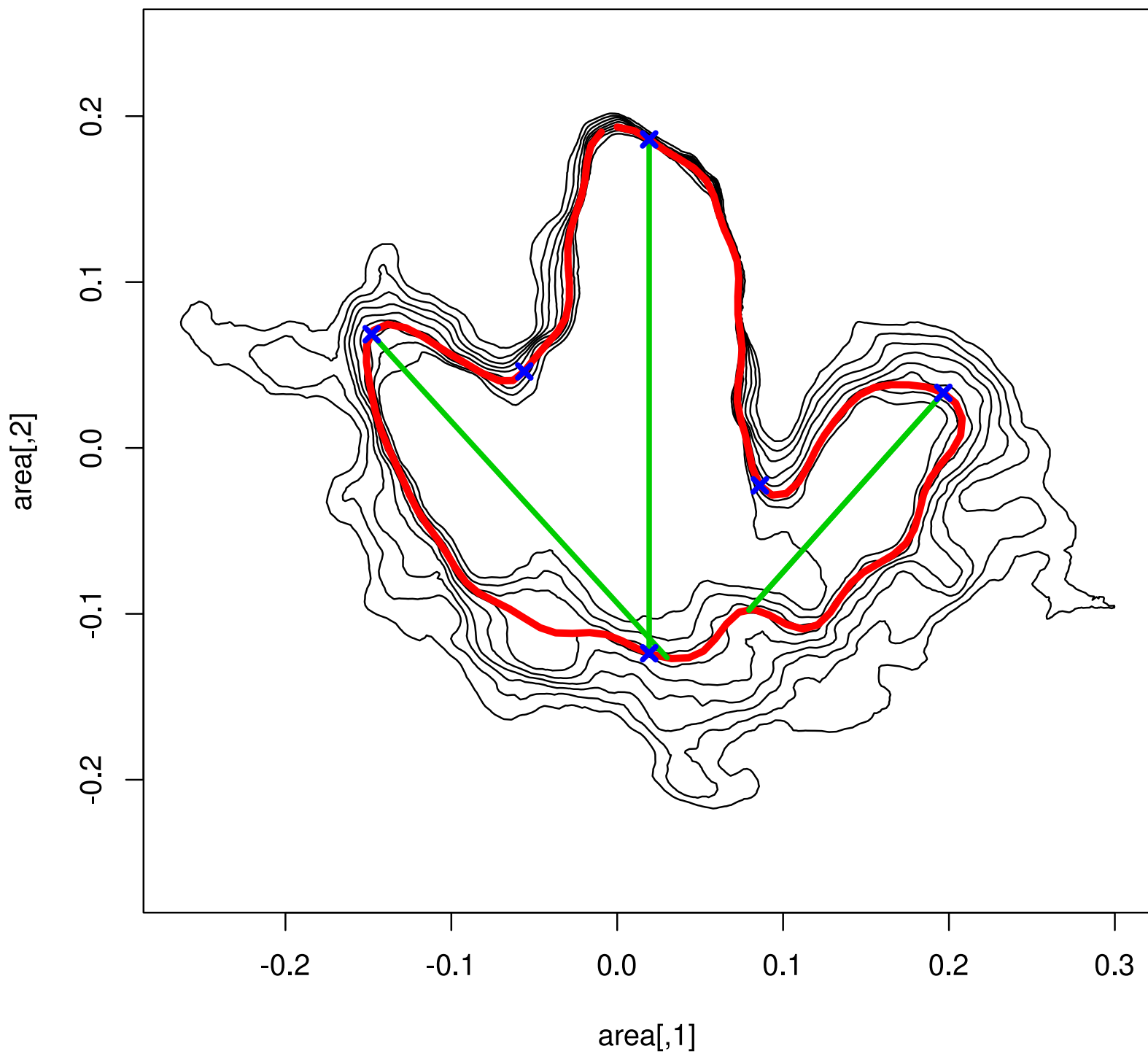

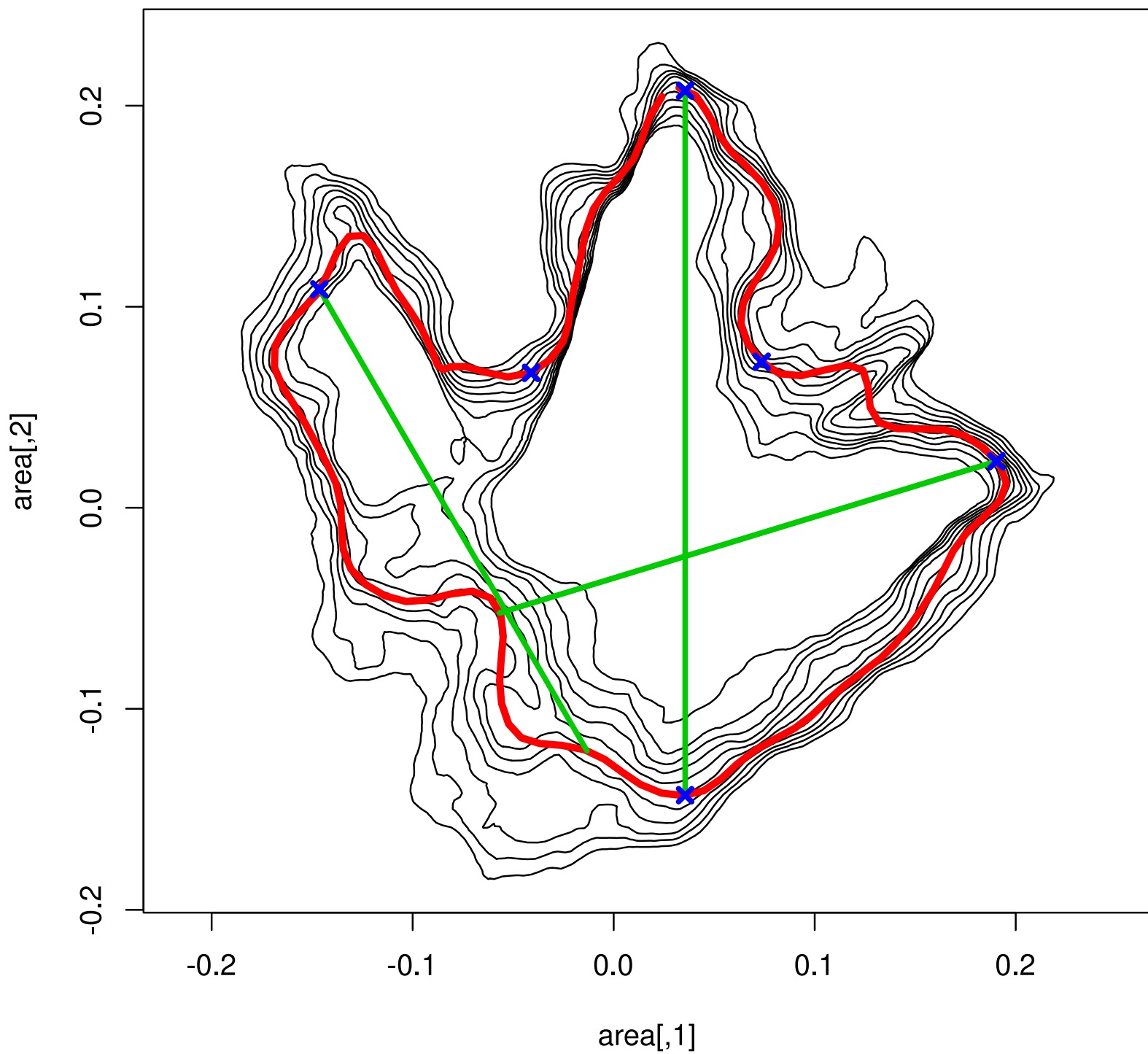

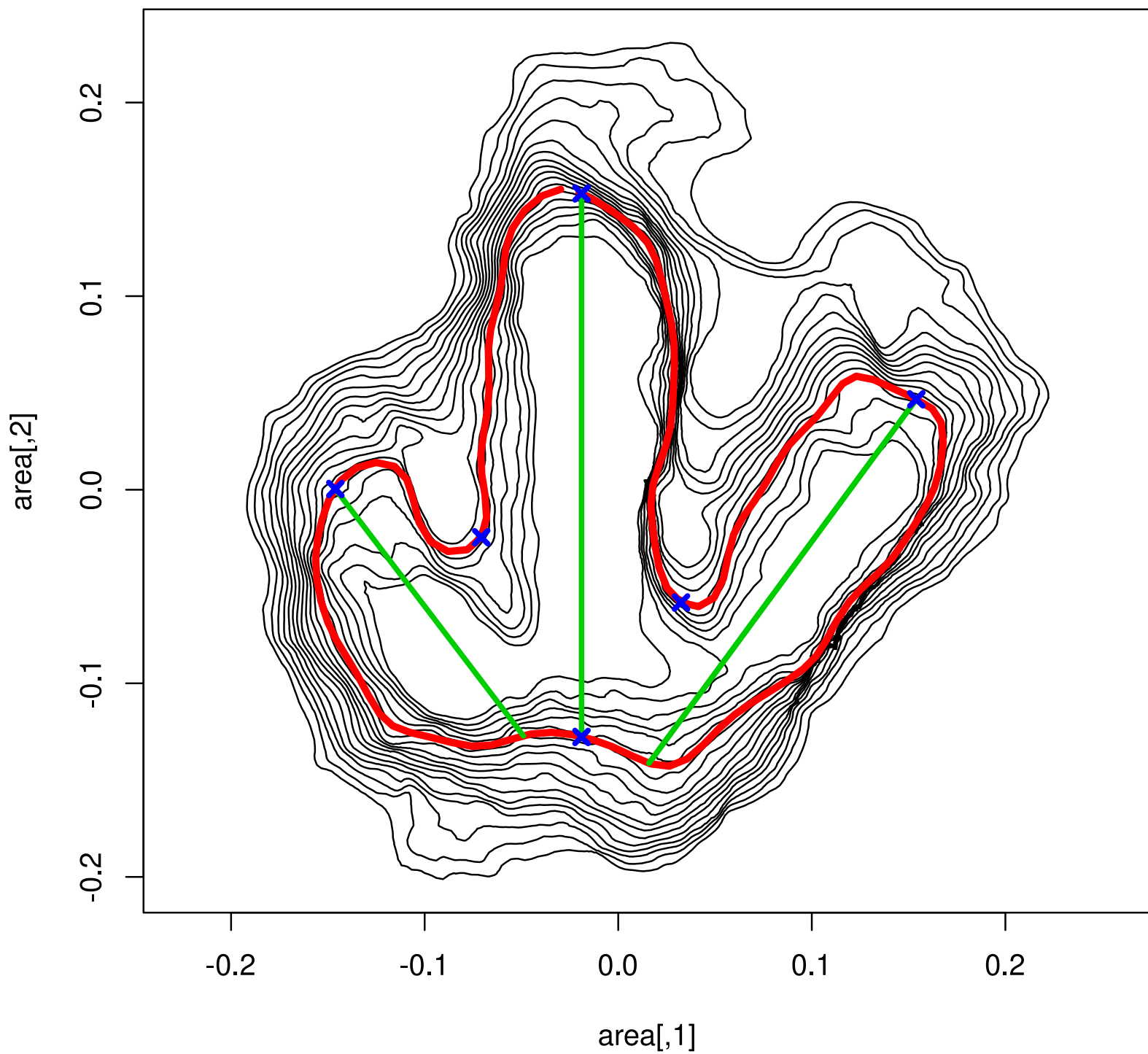

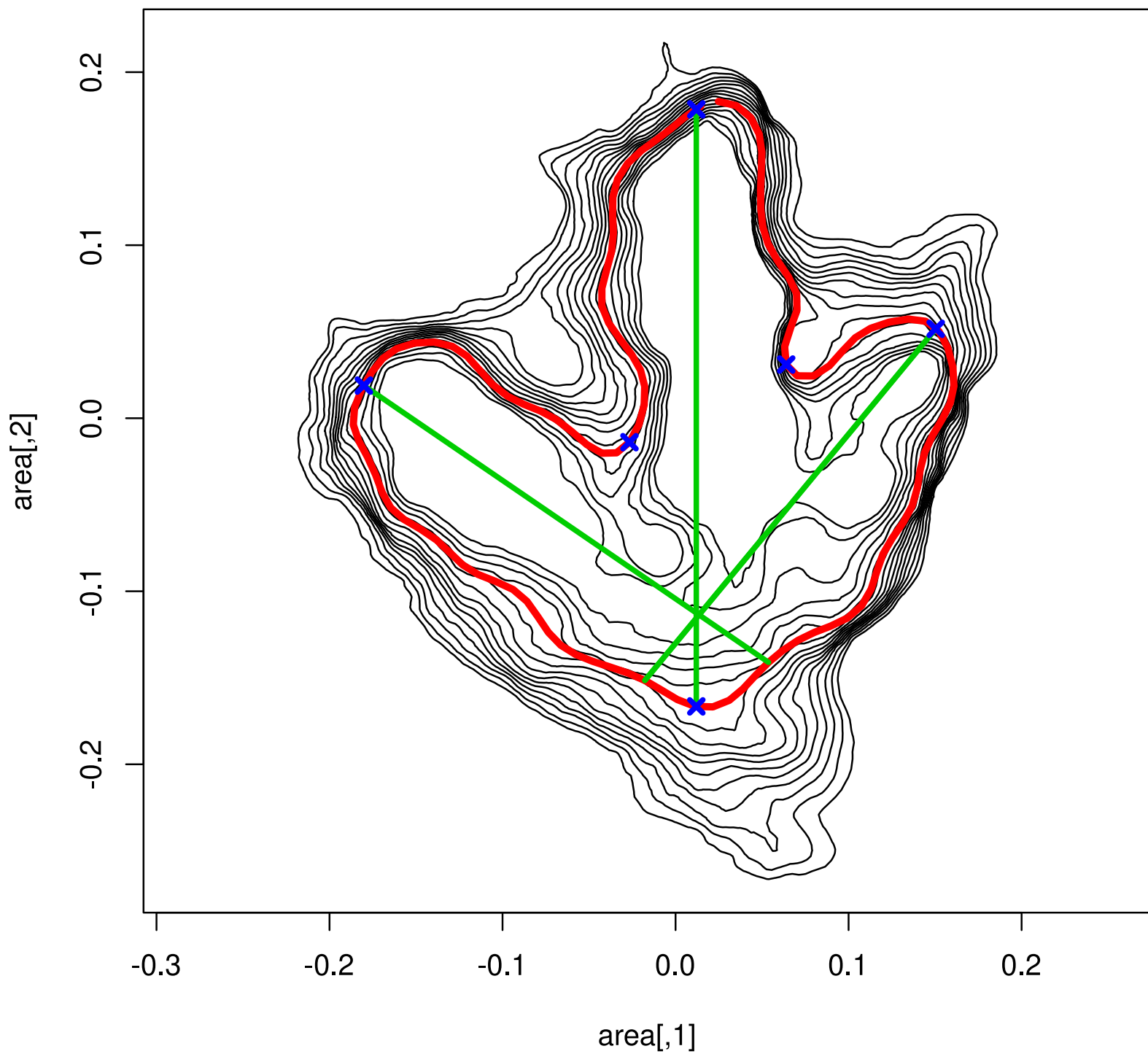

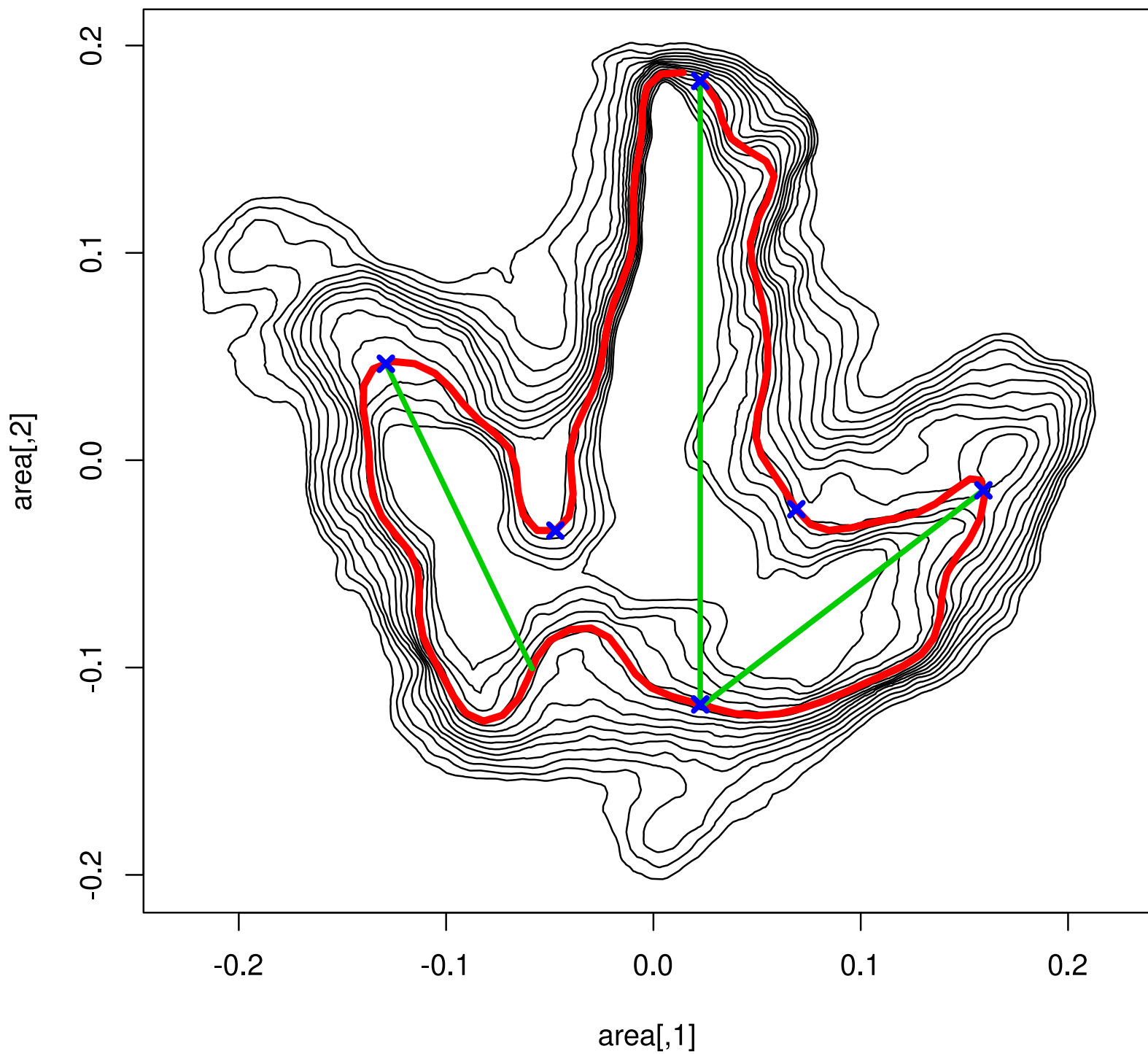

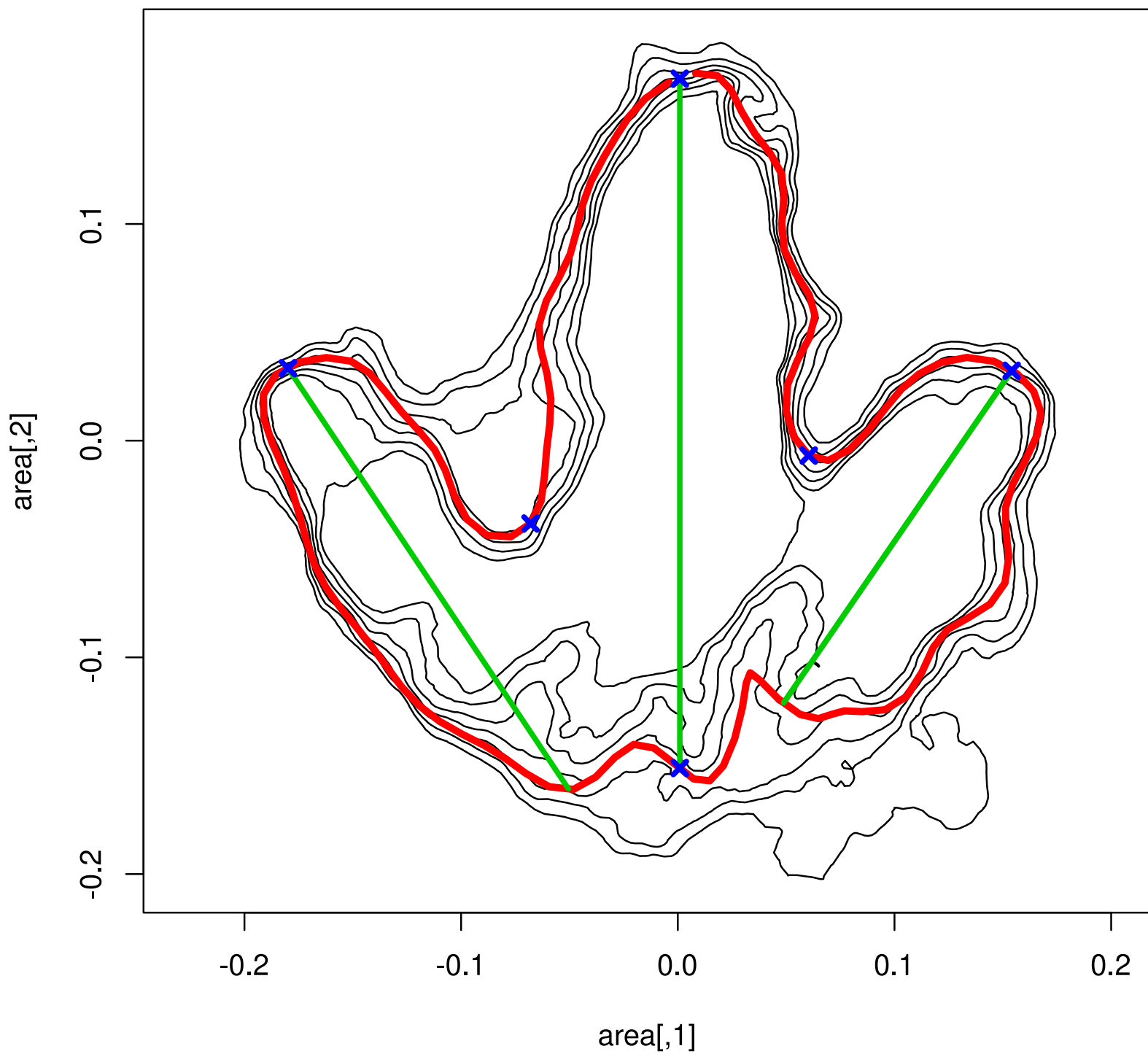

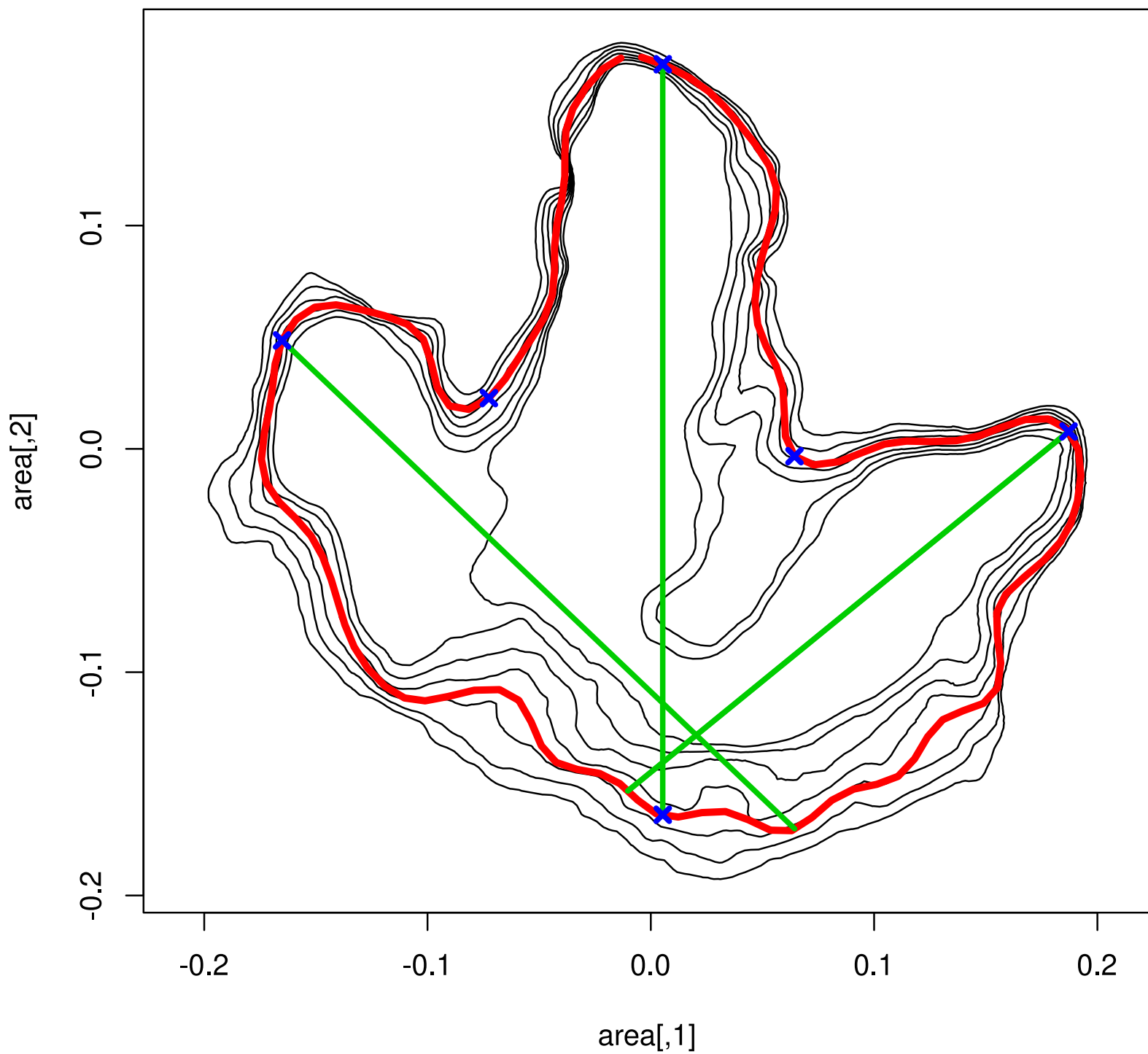

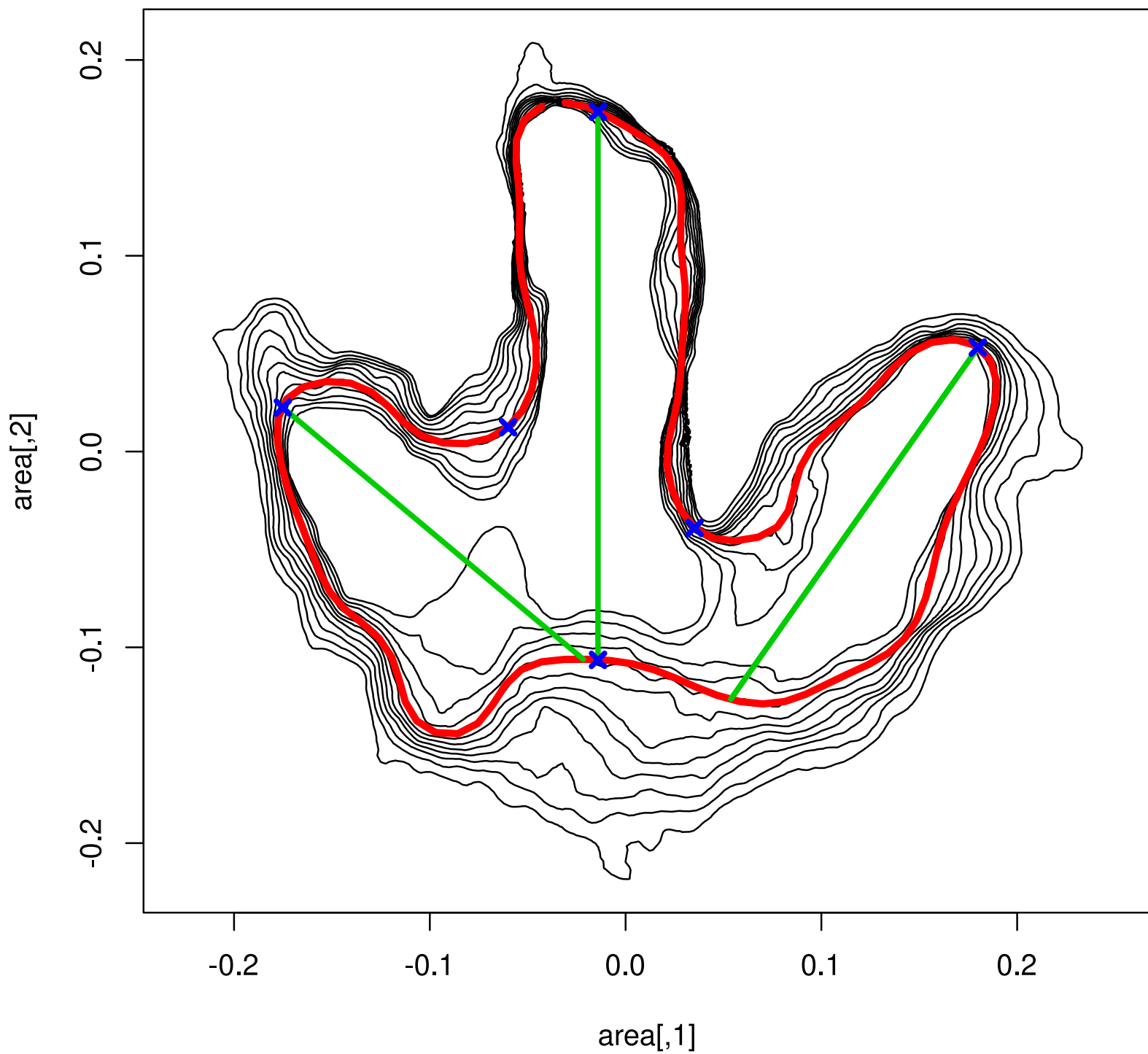

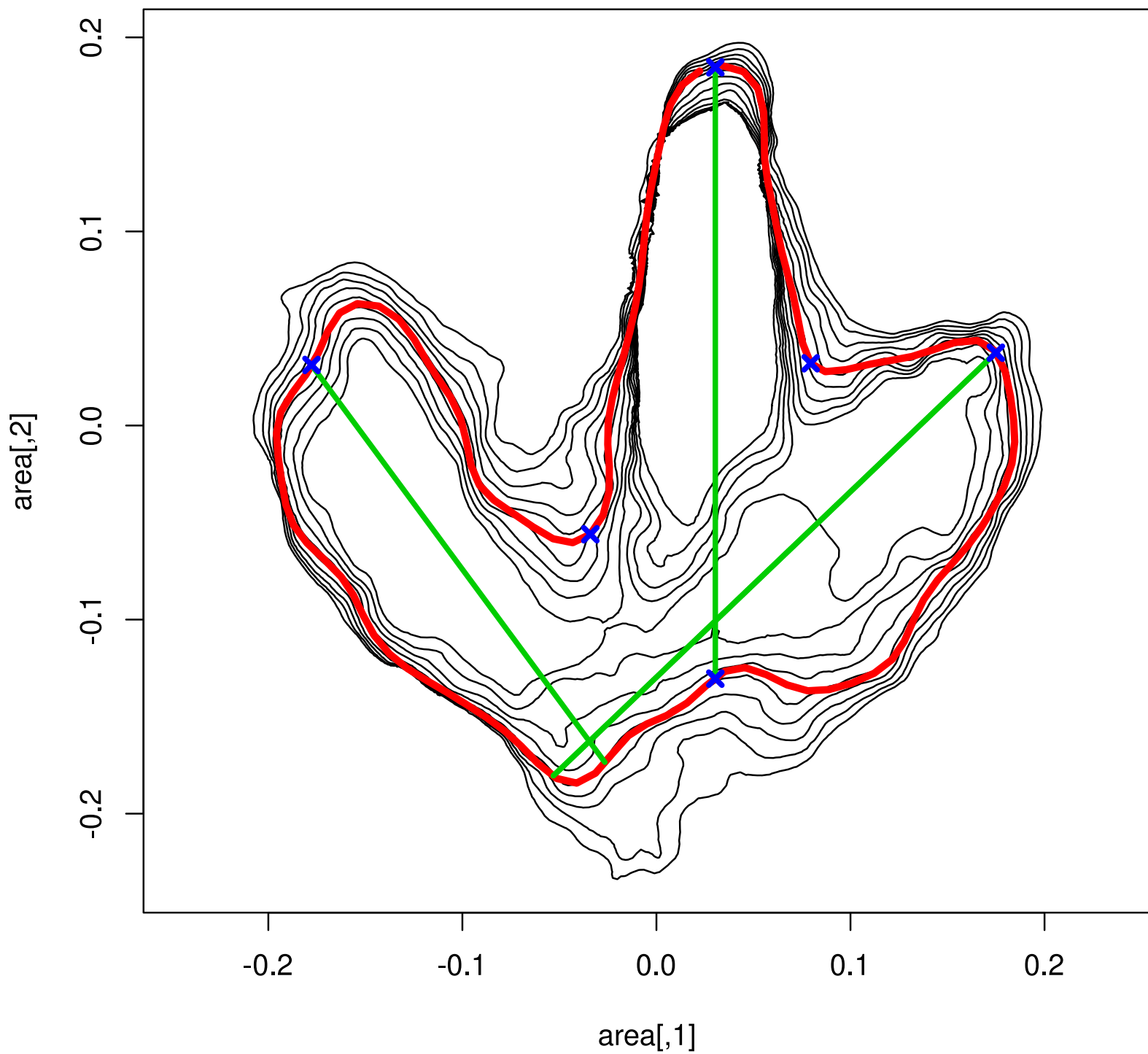

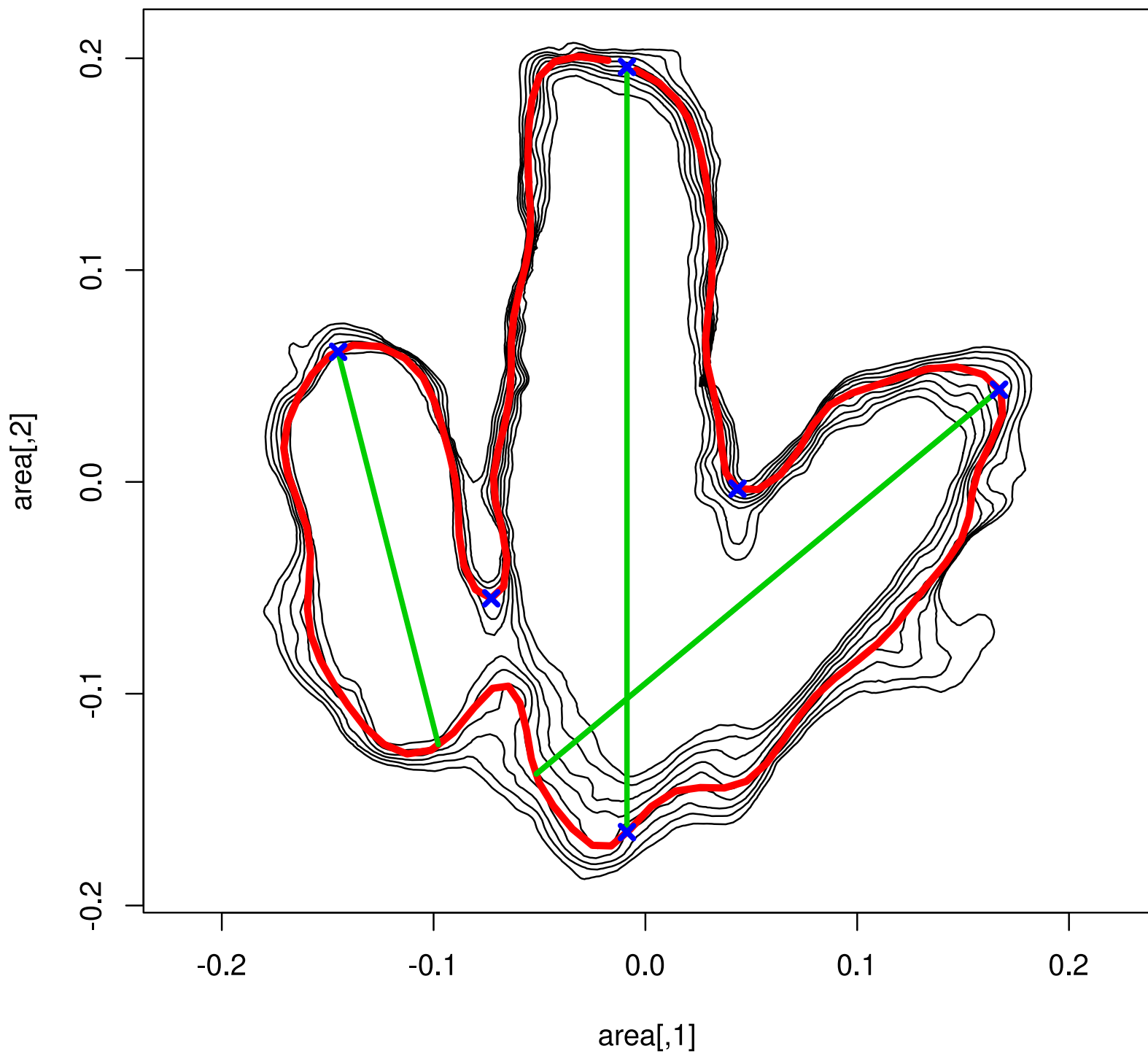

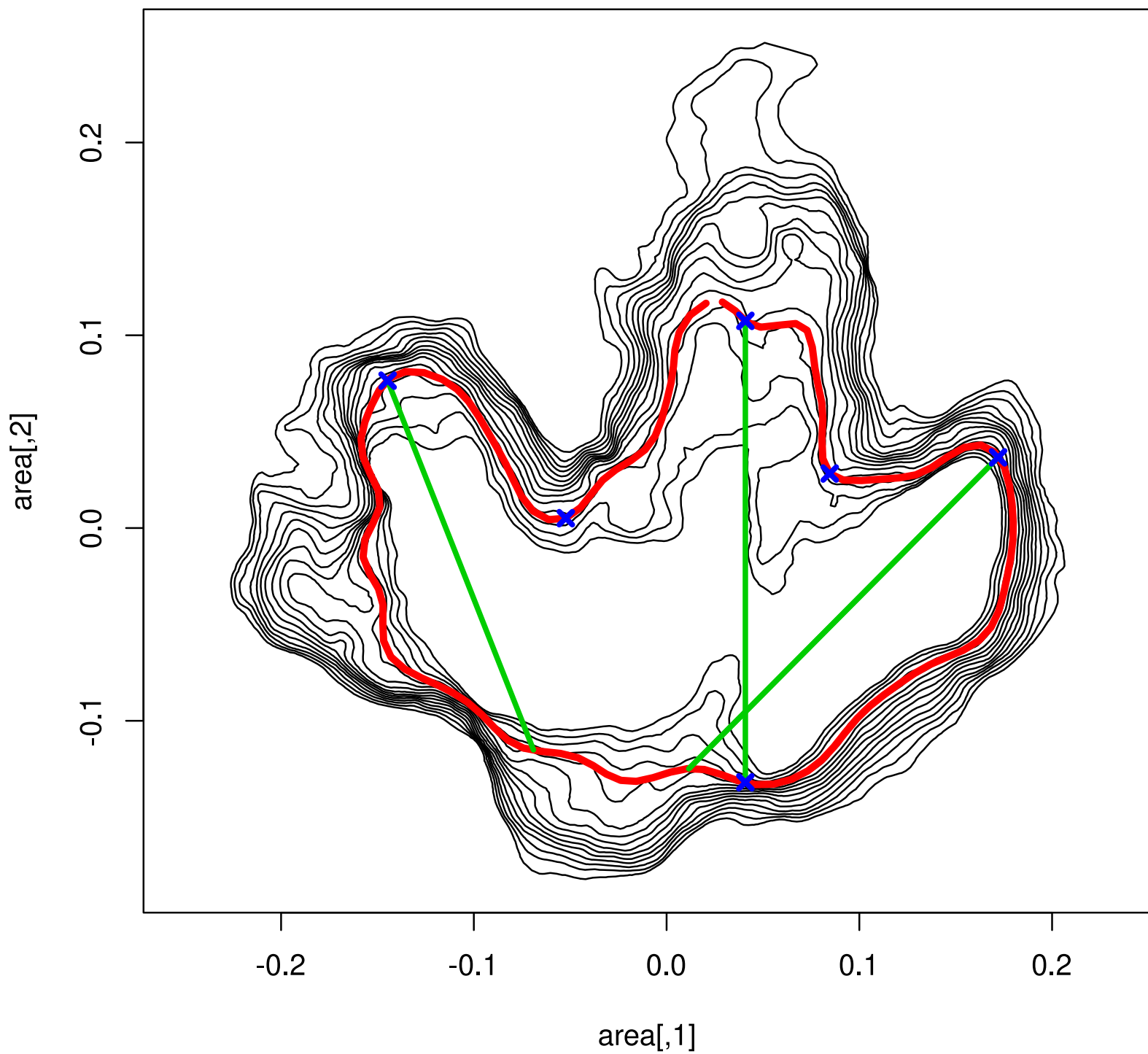

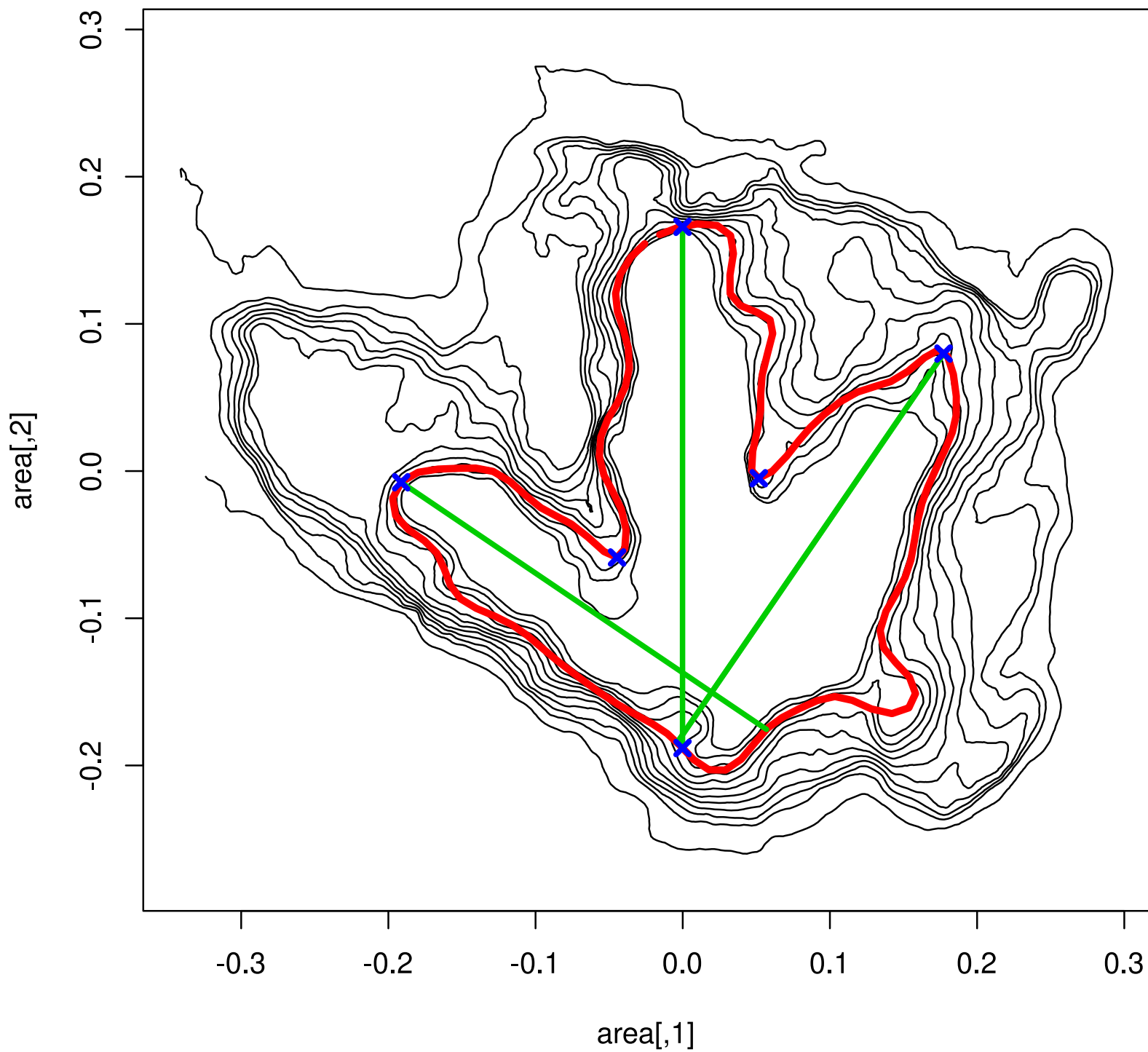

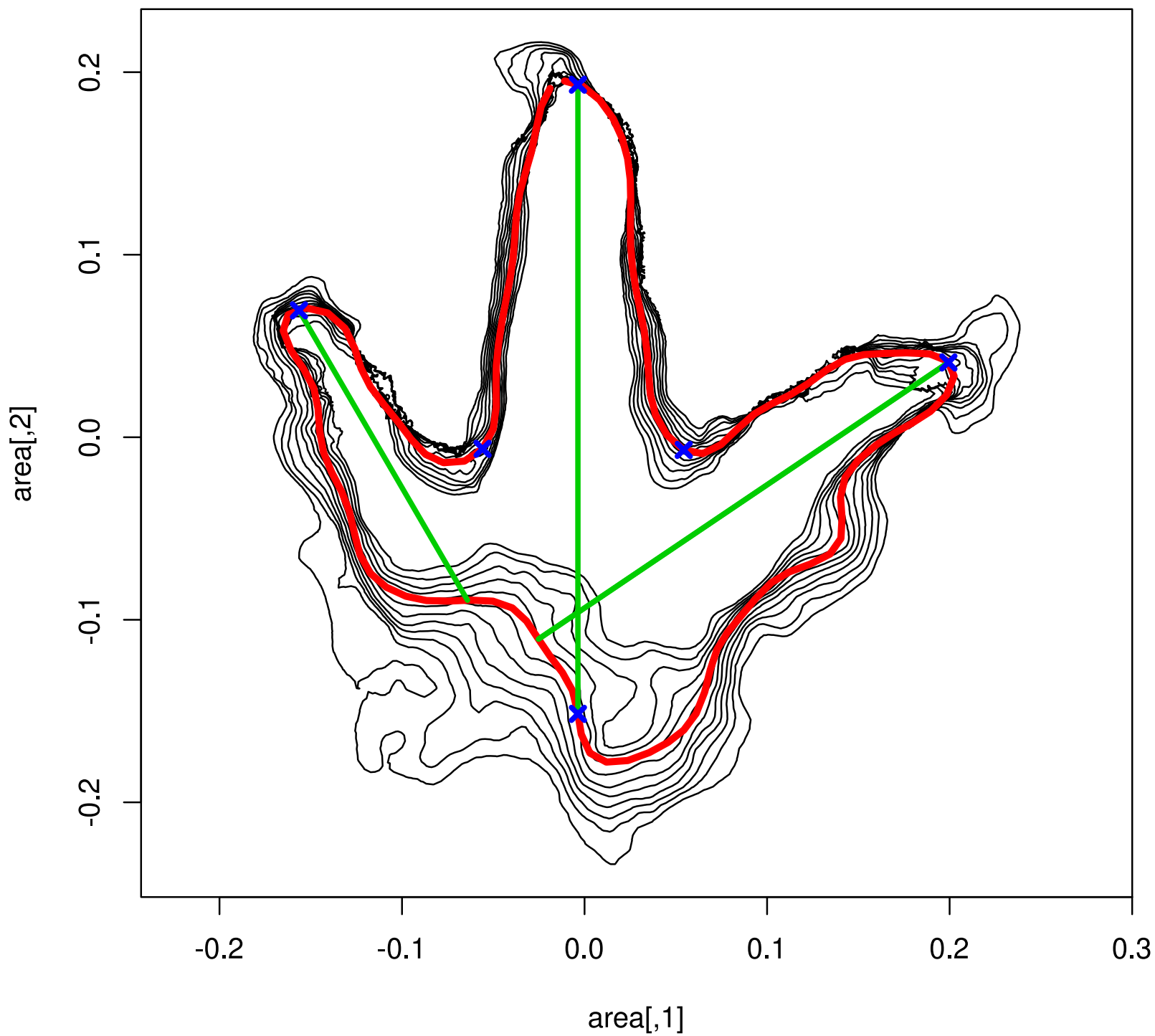

Supplement: File S3 [file peerj-07-7203-s003.pdf]
